# Macular evaluation of the retinal and choroidal vasculature changes in anterior ischemic optic neuropathy-a case control study

**DOI:** 10.1186/s12886-018-1007-8

**Published:** 2018-12-29

**Authors:** Hui Wang, Zhao-yang Meng, Song-guo Li, Jing-jing Wang, Jiao Sun, Hong-yang Li

**Affiliations:** 1 Department of Ophthalmology, Beijing Chaoyang Hospital, the Third Clinical Medical College of Capital Medical University, No. 8 Worker’s Stadium East Road, Chaoyang District, Beijing, 100043 People’s Republic of China; 2 Department of Ophthalmology, Beijing Friendship Hospital, the Second Clinical Medical College of Capital Medical University, No. 95 Yong’an Road, Xicheng District, Beijing, 100050 People’s Republic of China

**Keywords:** Nonarteritic anterior ischemic optic neuropathy, Vessel density, Choroid, Macular

## Abstract

**Background:**

This study aimes to characterize the fundus structural changes in patients with nonarteritic anterior ischemic optic neuropathy (NAION) and the correlation between macular vessel density, retinal nerve fibre layer (RNFL) parameters and visual field sensitivity (VFS) in NAION patients.

**Methods:**

A retrospective case control study was performed using 37 eyes with NAION, 30 uninvolved contralateral eyes, and 27 eyes of healthy age-matched subjects. Data on the retinas and choroidal vessel densities and VFS were compared among the three groups.

**Results:**

The NAION group exhibited significantly lower RNFL thicknesses, lower ganglion cell complexes (GCC), larger global loss volume (GLV) values and focal loss volume (FLV) values when compared with both uninvolved eyes and healthy eyes (*p* < 0.01 for all comparisons). The superficial vessel density (SVD) valus (whole, parafovea, superior-hemi and inferior-hemi) were significantly lower in NAION eyes, followed by uninvolved eyes and healthy eyes (*p* < 0.01; LSD, *p* < 0.05 for all comparisons). The deep vessel density (DVD) values (parafovea, superior-hemi and inferior-hemi) were the lowest by a significant value in NAION eyes, followed by uninvolved eyes and healthy eyes (*p* < 0.01; LSD, *p* < 0.05 for all comparisons). However, DVD value measurements (whole and fovea) of healthy and uninvolved eyes were not significantly different. The average threshold deviation (TD) was − 11.02 ± 3.75 dB for the overall field region, − 6.01 ± 2.21 dB for the affected superior field region and − 9.98 ± 3.34 dB for the affected inferior field region in NAION eyes. A statistically significant correlation was found between the RNFL thickness and visual field(VF) loss (*r* = − 0.788, *p* < 0.001).

**Conclusion:**

In addition to peripapillary vascular changes occurring in NAION eyes, macular vessel density is also involved. Furthermore, NAION-uninvolved eyes exhibited abnormalities compared with healthy eyes. This indicates that vascular changes may occur before changes in retinal thickness at the early stages of NAION.

**Electronic supplementary material:**

The online version of this article (10.1186/s12886-018-1007-8) contains supplementary material, which is available to authorized users.

## Background

Nonarteritic anterior ischemic optic neuropathy (NAION) is the most common acute optic neuropathy and severe visual impairment disease in individuals older than 50 years of age [1, 2]. The insufficient blood supply of short posterior ciliary arteries (PCA) may lead to anterior optic nerve hypoperfusion, which may cause the occurrence of NAION [3]. The underlying pathophysiology of NAION is unknown; however, it is presumed to be vascular in aetiology and caused by infarction of the retrolaminar portion of the optic nerve head (ONH), which is supplied by the short posterior ciliary arteries [4], although mechanical aetiologies have also been postulated [5].

The potential role of the microvasculature and vessel density in the pathophysiology of NAION has been extensively investigated. Histological studies on optic nerves associated with NAION have shown a complete loss of fibers in one half of the nerve and a peripheral loss of fibers or normal fiber expression in the other half [6]. Multiple studies have demonstrated correlation between visual field threshold and thinning of RNFL following an episode of optic neuritis or NAION [7]. Recently, increasing attention has been paid to the ONH in NAION diagnostics. Pasol [8] showed in their study that the retinal nerve fiber layer (RNFL) thickness was lower in optic neuropathies than controls. Previous studies have shown that C/D is lower in at risk eyes but becomes larger after NAION episode [9, 10]. Contreras et al. [11] found in their study that percentages of RNFL loss 3, 6, and 12 months after NAION onset were 38.9, 42.3, and 43.9%, respectively. Moreover, macular involvement was clearly demonstrated in patients with NAION [12]. Nevertheless, little research has been made on the blood flow and structural abnormalities in the macular area in NAION eyes. Although macula is less than 2% of the retina, it contains 30% of retinal ganglion cells (RGC).

Recently, the introduction of optical coherence tomography angiography (OCT-A) has allowed for acquisition of various retinal layers, providing a quantitative assessment of the microcirculation of various retinal diseases [13–17]. OCT-A had been used to observe the ONH vessel density in NAIONs. A previous study found that the peripheral vascular density of ONH in NAIONs decreased significantly [18]. Otherwise, the PCA supplies blood not only to the ONH but also to the choroid.

However, it remains uncertain what role, if any, the macular choroid plays in the pathogenesis of NAION despite some of the shared circulation from the posterior ciliary arteries. It is still unknown, if NAION eyes display abnormal blood flow as compared to the uninvolved fellow eyes and healthy eyes. In order to identify this role, OCTA tends to be more sensitive in detecting subtle changes in NAION eyes to observe whether or not NAIONs have abnormal blood flow before the onset of clinical symptoms. Our present study assesses the performance of OCT-A macular and retinal vessel density measurements for differentiating among healthy, uninvolved and NAION eyes.

## Methods

### Study design and participants

This retrospective case control study of healthy, uninvolved, and NAION eyes was performed at Beijing Chaoyang Hospital and Beijing Friendship Hospital, China. A total of 94 eyes were enrolled that completed OCT-A imaging and ONH imaging (AngioVue; Optovue, Inc., CA, USA) with good-quality images. The control group had 33 eyes of healthy age-matched subjects without any ophthalmic pathology. All patients diagnosed with NAION from January 2016 to March 2017 were considered for enrollment in this study. Subjects were divided into three groups: normal control, uninvolved, and NAION. Healthy eyes without NAION, fundus disease, or history of laser therapy or eye surgery were used as the normal controls. The uninvolved group included the 33 contralateral eyes not diagnosed with NAION.

NAION was diagnosed on the basis of some ophthalmologic examination, including detailed history, visual acuity assessment, optic nerve function tests, fundus examination, and visual field defects consistent with NAION. The time lag between the ischemic event and the OCT-A scan was at least 6 months to eliminate the effects of optic disk and RNFL edema observed in the acute phase.

The inclusion criteria were as follows [19]: (1) a history of sudden visual loss, usually discovered in the morning, which was not due to any ocular, systemic, or neurological diseases; (2) optic disk edema at the onset must have been documented in the Ocular Vascular Clinic or by another ophthalmologist; (3) spontaneous resolution of optic disk edema was observed; and (4) the eye had optic disk–related visual field defects.

The exclusion criteria were as follows [20]: (1) obvious retinopathy, including but not limited to high myopia and diabetic retinopathy; evidence of vitreoretinal diseases, such as polypoidal choroidal vasculopathy, ischemic central retinal vein occlusion, or diabetic retinopathy; retinal vascular lesions; medical history of fundus laser and intracranial injection of anti-vascular endothelial growth factor drug; (2) cataract prohibiting adequate retinal evaluation; (3) magnetic resonance imaging suggestive of demyelinating lesions in the brain or spinal cord; (4) evidence of arteritic ischemic optic neuropathy, such as history of scalp tenderness, a significantly elevated sedimentation rate, or elevated C-reactive protein level; (5) a history of inflammatory or infectious diseases (also eliminated by detailed history-taking or relative systemic and laboratory examination); (6) relapse of symptoms in the same eye, suggestive of idiopathic demyelinating optic neuritis; and (7) visual field defect larger than one hemifield, spherical equivalent ≥3D, and astigmatism ≥2D. Moreover, participants having an unreliable visual field, poor-quality OCT-A, or ONH scans were also excluded from this study.

The demographic and clinical characteristics including medical history of systemic disease were obtained from participants. Regular ophthalmic examinations and clinical examinations, including measurement of blood pressure (BP) and heart rate, collection of medical and ophthalmologic history, slit-lamp biomicroscopy, visual acuity assessment, intraocular pressure (IOP), dilated examination of fundus, and testing of visual field (VF). OCT-A images were obtained for all the patients by the same operator.

### Visual field acquisition

VFs were assessed with a commercial VF analyser (Humphrey Visual Field Analyzer; Carl Zeiss Meditech, Inc., Dublin, CA) using the Swedish interactive threshold algorithm (SITA) 24–2 Standard protocol. NAION patients with reliable VFs (defined by fixation losses with false-positive and false-negative results amounting to less than 33% of total results) were included in the study. Because superior and inferior field defects are the most common patterns seen in NAION, we calculated the average threshold deviation (TD) from normal for the superior and inferior regions. The patients were categorized into the following two groups according to locations of VF loss: “inferior field loss (IFL)” and “superior field loss (SFL)”. The VFs were averaged based on the decibel (dB) scale, with uniform weighting applied using measurement points.

### OCT-A image acquisition and processing

The AngioVue OCTA system featured in the commercially available Avanti SD-OCT device (RTVue-XR Avanti, Optovue, Fremont, CA) was used to capture the amplitude-decorrelation angiography images. The split-spectrum amplitude-decorrelation angiography (SSADA) algorithm (version 2015.100.0.35) was used to capture the dynamic motion of the red blood cells and to provide a high-resolution 3D visualization of perfused retinal vasculature. Using this software makes it possible to visualize the retinal and choroidal vasculature noninvasively via motion contrast. Vessel density was automatically calculated as the proportion of measured area occupied by flowing blood vessels defined as pixels having decorrelation values acquired by the SSADA algorithm above the threshold level. The motion correction technology software was used to remove saccades and minor loss of fixation [13].

**Fig. 1:**
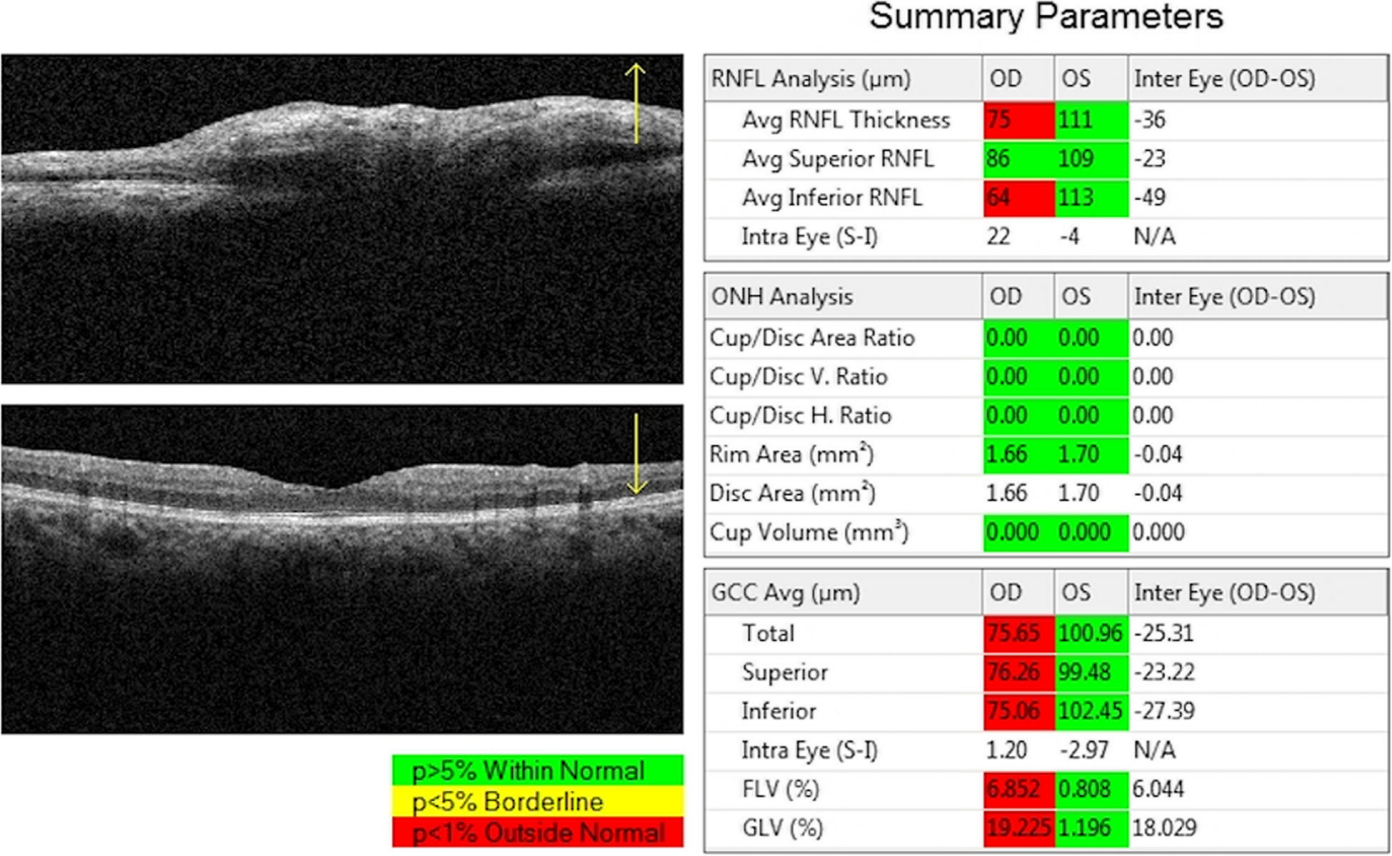
RTVue-100 OCT analysed the optic nerve head imaging in the optic disc area (ONH protocol, GCC protocol and 3D Disc) mode. The optic nerve head (ONH) map protocol was used to obtain RNFL thickness and ONH indices measurements; Other indices such as three avg. GCC parameters (total, superior and inferior), focal loss volume (FLV), and global loss volume (GLV) were measured in the GCC protocol

**Fig. 2:**
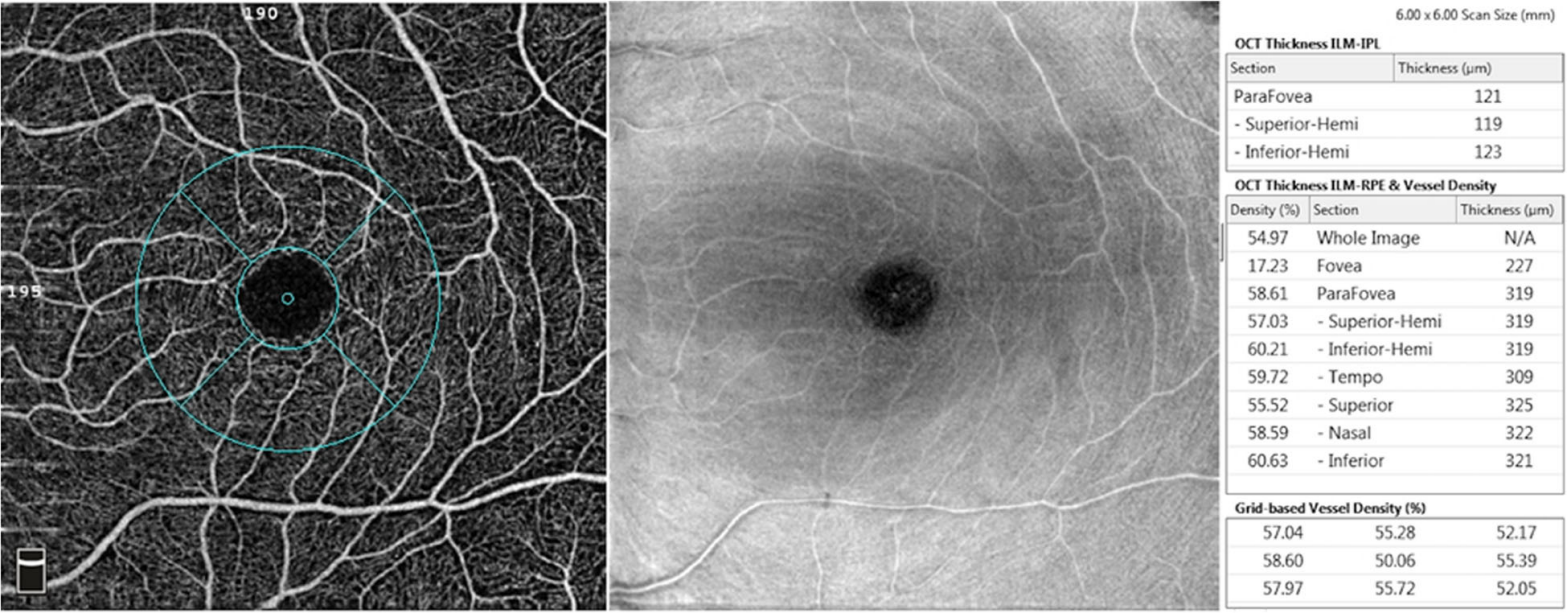
OCT angiogram of a superficial vascular capillary plexus in a 6 × 6 mm area centered on the macula. The en-face image of the superficial plexus was segmented with an inner boundary at 3 μm beneath the internal limiting membrane and the outer boundary was set at 15 μm beneath the inner plexiform layer

**Fig. 3:**
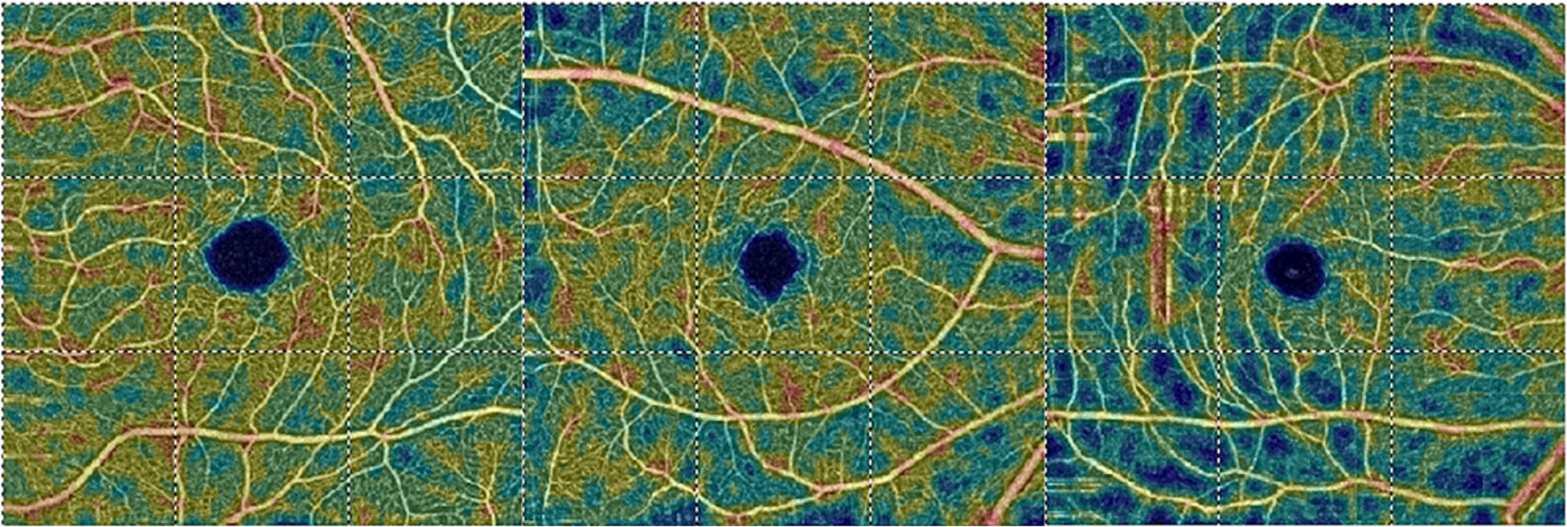
Retinal superficial vessel density map in healthy, uninvolved, and NAION eyes

OCT-A imaging was used to measure the superficial vessel density (SVD), deep vessel density (DVD), choroid vessel density (CVD), superficial retinal thickness (SRT) and deep retinal thickness (DRT) in the patients in vivo using the Angio Retina mode (6 × 6 mm^2^). The superficial retinal layer extends from the inner limiting membrane with an offset (from the interface reference) of 3 μm to the inner plexiform layer with an offset (from the interface reference) of 15 μm. The deep retinal layer was shown in the slab between 15 and 70 μm below the inner plexiform layer. Moreover, the ONH imaging [RNFL thickness and ONH and ganglion cell complex (GCC) indices measurements] was also analyzed using the RTVue-100 OCT in the optic disk area (ONH protocol, GCC protocol, and 3D Disk) in the tracking mode. The ONH map protocol was used to obtain the RNFL thickness and ONH indices. Other indices such as three average GCC parameters (total, superior, and inferior), focal loss volume (FLV), and global loss volume (GLV) were measured in the GCC protocol (Fig. 1, Fig. 2 and Fig. 3).

### Statistical analysis

The Kolmogorov–Smirnov test was used to identify the normality of distribution. Descriptive statistics were calculated as the mean and standard deviation for normally distributed variables and median, first quartile, and third quartile for nonnormally distributed variables. The categorical data were analyzed using the Fisher’s exact test. The one-way analysis of variance (ANOVA) test for normal distributions and Kruskal-Wallis tests for nonnormal distributions were used to compare other parameters between groups. The Tamhane’s T^2^ test was performed to adjust for multiple comparisons between groups within each analysis. Spearman rank correlation was used to assess correlations between VF variables and OCTA variables.

The diagnostic accuracy for differentiating between (1) healthy and NAION eyes and (2) healthy and uninvolved eyes were evaluated by calculating the area under the receiver operating characteristic (AUROC) curves. All reported *P* values were two sided. A *p* value < 0.05 was considered statistically significant. Statistical analysis was performed using the SPSS software version 21 (SPSS, Inc., IL, USA). Pairwise comparison of the AUROCs was performed using the method suggested by Pepe al. [21] to evaluate statistical significance between the receiver operating curves.

## Results

This study included 27 healthy eyes, 30 uninvolved eyes, and 37 NAION eyes with good-quality scans. The disease duration of the NAION patients was 9.25 ± 1.13 months. No statistically significant differences were found in age, gender, systolic, diastolic, mean BP, and mean ocular perfusion pressure measurements among the groups (Table 1).

**Table 1:** Demographic characteristics of all patients

Variables	Healthy	Uninvolved eyes	NAION eyes	*P* Value
	(*n* = 27)	(*n* = 30)	(*n* = 37)	
Demographic characteristics				
Age, year^b^	53.70 ± 9.95	58.17 ± 13.49	55.05 ± 7.93	*p* = 0.299
Gender, male/female	18/9	21/9	24/13	*p* = 0.905
Disease duration, months	–	–	9.25 ± 1.13	
Clinical characteristics				
Systolic BP, mm Hg^a^	128(120,130)	127(120,130)	120(115,130)	*p* = 0.148
Diastolic BP, mm Hg^a^	77(75,80)	80(80,80)	80(80,80)	*p* = 0.858
Heart rate, beats/min^b^	79.40 ± 5.91	79.53 ± 6.13	77.67 ± 4.53	*p* = 0.377
History of diabetes, n (%)	5(18.52%)	7(23.33%)	10(27.03%)	*p* = 0.730
History of hypertension, n (%)	6(22.22%)	7(23.33%)	9(24.32%)	*p* = 0.981

*BP* Blood pressure, *NAION* nonarteritic anterior ischemic optic neuropathy

Statistical significance tested by ANOVA for normal distributions and Kruskal–Wallis tests for nonnormal distributions; all comparisons were corrected with the post hoc test. The categorical data were analyzed using the Fisher’s exact test of chi-square tests

^a^Nonnormally distributed variables, represented by median (interquartile range)

^b^Normally distributed variables, represented by mean (±standard deviation)

### Ocular and optic nerve characteristics of all the patients

IOP was not significantly different among the three groups (*p* = 0.454). The pairwise comparisons of RNFL showed that NAION eyes (70.86 ± 10.50 μm) had significantly lower RNFL thickness values compared with both uninvolved eyes (98.90 ± 10.55 μm) and healthy eyes (102.11 ± 7.21 μm) [least significant difference (LSD), *p* < 0.001 for both]. However, the RNFL thickness values was not significantly different between uninvolved and healthy eyes (*p* = 0.215).

Statistically significant differences were found among the three groups. NAION eyes (72.15 ± 10.68 μm; 24.02% ± 8.75%) had significantly lower GCC thickness values and larger GLV values compared with both uninvolved eyes (96.36 ± 6.82 μm; 4.56% ± 3.60%) and healthy eyes (98.27 ± 7.43 μm; 3.72% ± 1.70%) (LSD, *p* < 0.001 for both), but no significant difference was found between uninvolved and healthy eyes (*p* = 0.408 and 0.596, respectively). The FLV values were significantly different among the three groups (*p* < 0.001). NAION eyes (9.59% ± 3.34%) had significantly larger FLV values compared with both uninvolved eyes (2.11% ± 1.65%) and healthy eyes (0.86% ± 0.40%); all three pairwise comparisons were statistically significant (LSD, *p* < 0.05 for all comparisons).

The visual field deficits varied, but were typical of NAION. The average TD was − 11.02 ± 3.75 dB for overall field region, − 6.01 ± 2.21 dB for the affected superior field region and − 9.98 ± 3.34 dB for the affected inferior field region in NAION eyes (Table 2).

**Table 2:** Optic nerve characteristics and visual performance of all the patients

Variables	Healthy eyes	Uninvolved eyes	NAION eyes	*P* Value
	(n = 27)	(n = 30)	(n = 37)	
Ocular characteristics				
IOP, mm Hg	13.99 ± 2.90	14.31 ± 2.00	14.90 ± 2.50	*p* = 0.454
RNFL Thickness (μm)	102.11 ± 7.21	98.90 ± 10.55	70.86 ± 10.50	*p* < 0.001^*,‡,§^
GCC Analysis				
GCC Thickness (μm)	98.27 ± 7.43	96.36 ± 6.82	72.15 ± 10.68	*p* < 0.001^*,‡,§^
FLV (%)	0.86 ± 0.40	2.11 ± 1.65	9.59 ± 3.34	*p* < 0.001^*,†,‡,§^
GLV (%)	3.72 ± 1.70	4.56 ± 3.60	24.02 ± 8.75	*p* < 0.001^*,‡,§^
VF parameter (Mean ± SD, dB)				
MD	N/A	N/A	−11.02 ± 3.75	–
Superior TD	N/A	N/A	−6.01 ± 2.21	–
Inferior TD	N/A	N/A	−9.98 ± 3.34	–

*FLV* Focal loss volume, *GCC* ganglion cell complex, *GLV* global loss volume, *IOP* intraocular pressure, *NAION* nonarteritic anterior ischemic optic neuropathy, *RNFL* retinal nerve fiber layer, *VF* visual field, *MD* mean deviation, *dB* decibels

^*^Statistical significance tested by ANOVA for normal distributions and Kruskal-Wallis tests for nonnormal distributions, all comparisons were corrected with post hoc test

^†^ Significant difference between mean values of healthy eyes and uninvolved eyes

^‡^ Significant difference between mean values of healthy eyes and NAION eyes

^§^ Significant difference between mean values of uninvolved eyes and NAION eyes

### OCT-A parameters of all the patients

The ANOVA test of SVD value measurements showed that the vessel density values were significantly different among the three groups (*p* < 0.001). The whole, parafovea, superior-hemi and inferior-hemi values were significantly lower in NAION eyes (44.31, 48.76, 48.72 and 49.05%, respectively), followed by uninvolved eyes (50.55, 54.37, 53.98 and 54.74%, respectively) and healthy eyes (54.50, 56.57, 56.37 and 56.10%, respectively). All three pairwise comparisons were statistically significant (LSD, *p* < 0.05 for all comparisons). The deep vessel density (DVD) value measurements (the parafovea, superior-hemi, and inferior-hemi vessel density values) were significantly lower in NAION eyes (54.36% ± 3.36, 54.87% ± 3.97, 53.95% ± 3.80%, respectively), followed by uninvolved eyes (59.09% ± 1.97, 59.05% ± 2.23, 59.13% ± 1.97%, respectively) and healthy eyes (62.08% ± 1.25, 61.82% ± 1.94, 60.75% ± 2.23%, respectively). All three pairwise comparisons were statistically significant (LSD, *p* < 0.05 for all comparisons).

The choroid vessel density (CVD) values were significantly different among the three groups (*p* < 0.001) except for the whole and fovea quarters. The comparisons also showed that NAION eyes had lower whole vessel density compared with healthy eyes (*p* = 0.001, 0.006, and 0.036, respectively). The aforementioned vessel density values were significantly lower in NAION eyes than in uninvolved eyes (*p* < 0.05); however, no significant differences were found between uninvolved and healthy controls.

The comparison of superficial retinal thickness (SRT) values showed that NAION eyes (96.43 ± 14.40 μm; 95.43 ± 13.67 μm; 96.59 ± 15.65 μm) had significantly lower parafovea retinal thickness values (96.43 ± 14.40 μm) compared with both uninvolved eyes (111.47 ± 12.14 μm) and healthy eyes (111.89 ± 11.86 μm) (*p* < 0.001); the comparison of deep retinal thickness (DRT) values showed that NAION eyes had significantly lower parafovea retinal thickness values (183.32 ± 11.86 μm) compared with both uninvolved (191.67 ± 12.31 μm) and healthy eyes (193.04 ± 9.37 μm) (*p* = 0.001). However, no significant differences were observed between uninvolved and healthy eyes (*p* = 0.903 and 0.650, respectively). (Table 3).

**Table 3:** Mean vessel density and retinal thickness measured in OCT angiography of all the patients

OCTA Parameters	Healthy	Uninvolved eyes	NAION eyes	*P* Value
	(n = 27)	(n = 30)	(n = 37)	
SVD (%)				
Whole	54.50 ± 1.34	50.55 ± 3.27	44.31 ± 5.35	*p* < 0.001^*,†,‡,§^
Fovea	29.57 ± 1.68	25.40 ± 5.76	23.28 ± 5.08	*p* < 0.001^*,†,‡^
Parafovea	56.57 ± 1.18	54.37 ± 3.13	48.76 ± 5.17	*p* < 0.001^*,†,‡,§^
Superior-Hemi	56.37 ± 1.72	53.98 ± 3.55	48.72 ± 5.50	*p* < 0.001^*,†,‡,§^
Inferior -Hemi	56.73 ± 1.51	54.74 ± 3.20	49.05 ± 5.07	*p* < 0.001^*,†,‡,§^
DVD (%)				
Whole	60.35 ± 1.12	60.08 ± 1.77	57.12 ± 2.48	*p* < 0.001^*,‡,§^
Fovea	31.49 ± 2.21	30.36 ± 3.67	26.73 ± 2.70	*p* < 0.001^*,‡,§^
Parafovea	62.08 ± 1.25	59.09 ± 1.97	54.36 ± 3.36	*p* < 0.001^*,†,‡,§^
Superior-Hemi	61.82 ± 1.94	59.05 ± 2.23	54.87 ± 3.97	*p* < 0.001^*,†,‡,§^
Inferior -Hemi	60.75 ± 2.23	59.13 ± 1.97	53.95 ± 3.80	*p* < 0.001^*,†,‡,§^
CVD (%)				
Whole	64.12 ± 0.95	63.94 ± 1.49	63.33 ± 1.27	*p* = 0.035^*,‡^
Fovea	59.39 ± 1.94	58.78 ± 2.90	59.07 ± 4.09	*p* = 0.775^*^
Parafovea	64.97 ± 1.34	65.68 ± 1.50	63.21 ± 2.26	*p* < 0.001^*,‡,§^
Superior-Hemi	66.13 ± 1.65	65.64 ± 1.76	62.90 ± 2.34	*p* < 0.001^*,‡,§^
Inferior -Hemi	65.56 ± 1.49	65.73 ± 1.59	63.15 ± 1.80	*p* < 0.001^*,‡,§^
Parafovea SRT (um)	111.89 ± 11.86	111.47 ± 12.14	96.43 ± 14.40	*p* < 0.001^*,‡,§^
Parafovea DRT (um)	193.04 ± 9.37	191.67 ± 12.31	183.32 ± 11.86	*p* = 0.001^*,‡,§^

*NAION* Nonarteritic anterior ischemic optic neuropathy, *OCT-A* optical coherence tomography angiography, *SVD* superficial vessel density, *DVD* deep vessel density, *CVD* choroid vessel density, *SRT* superficial retinal thickness, *DRT* deep retinal thickness

^*^Statistical significance tested by ANOVA for normal distributions and Kruskal–Wallis tests for nonnormal distributions; all comparisons were corrected with the post hoc test

^†^Significant difference between mean values of healthy eyes and uninvolved eyes

^‡^Significant difference between mean values of healthy eyes and NAION eyes

^§^Significant difference between mean values of uninvolved eyes and NAION eyes

### Spearman rank correlation analyses on structural variables and visual field sensitivity

There was a statistically significant correlation between the RNFL thickness and VF loss (*r* = − 0.788, *s* < 0.001). However, the SVD; DVD and CVD values were not significantly associated with VF loss (Table 4).

**Table 4:** Spearman rank correlation analyses on RNFL thickness, vessel density and visual field

Variables	VF loss	
	Spearman rank correlation	
	r	*p*
RNFL thickness (mm)	−0.788	< 0.001
Whole SVD (%)	−0.355	0.372
Whole DVD (%)	−0.417	0.128
Whole CVD (%)	−0.265	0.108

*RNFL* retinal nerve fiber layer, *SVD* superficial vessel density, *DVD* deep vessel density, *CVD* choroid vessel density, *VF* visual field

### Diagnostic accuracy (AUROC)

The AUROC curve for differentiating between healthy and NAION eyes was the highest for average RNFL thickness (0.99), followed by whole SVD (0.96), whole DVD (0.89), parafovea SRT (0.81), parafovea DRT (0.73), and whole DVD (0.68). Their diagnostic accuracies in whole vessel density (0.90), parafovea vessel density (0.86), and fovea vessel density (0.82) were lower compared with RNFL thickness (0.96) (*p* < 0.01 for all). Pairwise comparisons showed that the AUROC of whole vessel density (0.90) was significantly higher compared with fovea vessel density (0.82) (*p* < 0.05), but similar to parafovea vessel density (0.86) (*p* > 0.05) (Table 5).

**Table 5:** Diagnostic accuracy (AUROC) for OCT-A vessel density and RNFL thickness measurements in all the patients

Diagnostic parameters	Healthy eyes	Uninvolved eyes	NAION eyes	*p* Value	AUROC (SE) NAION vs. healthy Eyes	AUROC (SE) Uninvolved eyes vs. healthy Eyes
	(95% CI)	(95% CI)	(95% CI)			
Average RNFL, um	102.1 (99.6–105.1)	98.9 (96.0–103.1)	70.9 (67.7–74.1)	< 0.001^*,†,‡^	0.99 (0.01)	0.68 (0.07)
Parafovea SRT, um	111.9 (107.7–115.8)	111.5 (107.3–115.3)	96.4 (92.3–101.0)	< 0.001^*,†,‡^	0.81 (0.05)	0.50 (0.08)
Parafovea DRT, um	193.0 (189.5–197.3)	191.7 (187.6–196.1)	183.3 (179.7–187.3)	0.001^*,†,‡^	0.73 (0.06)	0.51 (0.08)
OCT-A whole SVD,%	54.5 (54.0–55.0)	50.5 (49.5–51.8)	44.3 (42.5–46.0)	< 0.001^*,†,,‡§^	0.96 (0.03)	0.89 (0.05)
OCT-A whole DVD,%	60.3 (59.9–60.8)	60.1 (59.6–60.6)	57.1 (56.1–57.8)	< 0.001^*,†,‡,§^	0.89 (0.04)	0.53 (0.08)
OCT-A whole CVD,%	64.1 (63.8–64.5)	63.9 (63.4–64.5)	63.3 (62.9–63.7)	0.079	0.68 (0.07)	0.53 (0.08)

*AUROC* Area under the receiver operating characteristic, *CI* confidence interval, *CVD* choroid vessel density, *DRT* deep retinal thickness, *DVD* deep vessel density, *NAION* Nonarteritic anterior ischemic optic neuropathy, *OCT-A* optical coherence tomography angiography, *SE* standard error, *SRT* superficial retinal thickness, *SVD* superficial vessel density, *RNFL* retinal nerve fiber layer

^*^Statistical significance tested by ANOVA; corrected with the post hoc test

^†^Significant difference between mean values of healthy eyes and uninvolved eyes

^‡^Significant difference between mean values of uninvolved and NAION eyes

^§^Significant difference between mean values of healthy and uninvolved eyes

**Fig. 4:**
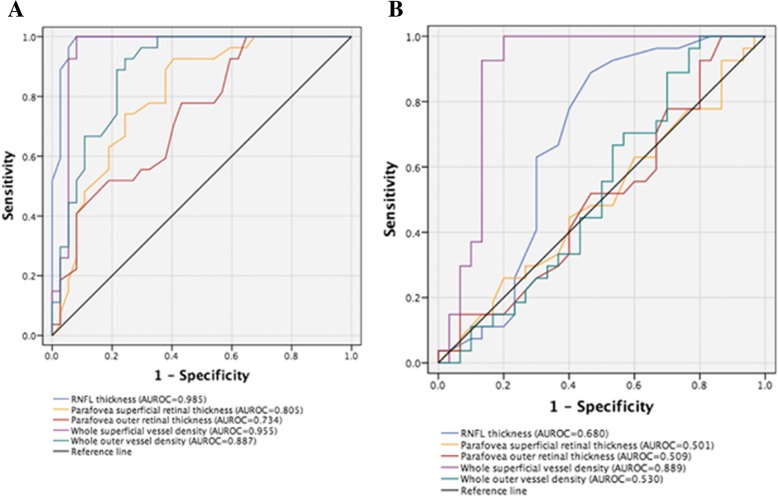
**a**. Area under the receiver operator characteristic curves for average RNFL thickness (0.99), followed by whole SVD (0.96), whole DVD (0.89), parafovea SRT (0.81), and parafovea DRT (0.73), for differentiating between healthy and NAION eyes. **b**. Area under the receiver operator characteristic curves for average whole SVD (0.89), followed by average RNFL thickness (0.68) for differentiating between healthy and uninvolved eyes

The AUROC curve for differentiating uninvolved eyes from healthy eyes was the highest for average whole SVD (0.89), followed by average RNFL thickness (0.68) (Fig. 4).

## Discussion

NAION is the most common and visually threatening optic nerve disorder in the middle-aged and elderly population. The present study demonstrated that the OCT-A vessel density measured in the superficial and deep layers of the retina distinguishes among groups of healthy, uninvolved, and NAION participants. Decreased retinal perfusion was found in NAION eyes, a finding that was correlated with retinal thinning. This study was novel in using OCT-A to demonstrate the retinal perfusion changes in optic atrophy after NAION.

OCT has been shown to be useful in quantifying peripapillary RNFL thickness in NAION [22]. It is also an approximation of the actual RGC axon loss because the RNFL contains glia and blood vessels, which may undergo concomitant changes related to ischemia. RNFL loss in NAION patients has been described in several studies. Rebolleda et al. [23] evaluated the anatomic outcomes after systemic steroid treatment in NAION and found no differences when comparing average RNFL loss with un-treated patients. RNFL thickness was significantly lower in NAION patients compared with the uninvolved eyes [24]. In this study, a significant RNFL loss in eyes were found with NAION (70.86 ± 10.50 μm) compared with both uninvolved eyes (98.90 ± 10.55 μm) and healthy eyes (102.11 ± 7.21 μm), consistent with previous findings. The reason for this change was due to the progressive RNFL thinning as the disease progressed toward optic atrophy described by Savini et al. [22]. However, no significant difference was found between uninvolved and healthy eyes.

VF defects are also hallmarks of NAION. Multiple studies have demonstrated correlation between VF threshold and RNFL thinning following episodes of optic neuritis or NAION. Danesh-Meyer et al. [25] and Hood et al. [26] observed a thinner NFL in NAION compared with the controls and correlation between the severity of VF loss and peripapillary NFL loss. Our study confirms these previous observations and shows a significant correlation between thinner RNFLs and VF loss in NAION patients. Kupersmith et al. [7] found in their study that abnormal RNFL birefringence occurs in sectors corresponding to regional VF loss during acute NAION when OCT-derived RNFL shows thickening. This contradicts the above result, possibly because the patients in this study were all in the acute phase of NAION, OCT showing thickening of the RNFL during acute optic nerve edema.

Previous studies about NAION hemodynamic changes focused on the circulatory insufficiency in the ONH; peripapillary retinal perfusion was significantly decreased in optic atrophy after NAION [27]. OCT-A may aid in the understanding of structure–function–perfusion relationships in NAION [27]. Little research was available on macular blood flow in patients with NAION. In this study, the vessel density and retinal thickness both in superficial and deep layers were found to be significantly different among the three groups. OCT-A SVT, DVD, SRT, and DRT were significantly lower in NAION patients compared with fellow uninvolved eyes. Circulatory insufficiency in the ONH is a widely accepted cause of NAION, which is associated with the dysfunction of short posterior ciliary arteries. The circulatory insufficiency has been found more often in the unaffected eye [20, 28]. Short posterior ciliary arteries can provide blood supply to the outer layers of retina; the insufficient blood perfusion of short posterior ciliary arteries may result in alterations in the blood supply of the outer layers of retina, which was reflected in this study. Although the mechanism of this outcome is not clear at the moment, the changes in the parameters can provide some evidence in diagnosing NAION. In this study, we also found that the vessel density in the superficial layer decreased in both NAION and uninvolved eyes compared with that of the normal control group of subjects without NAION. Gonul et al. [29] found a thinner subfoveal choroidal thickness (SCT) in both NAION and contralateral eyes in comparison to normal eyes after adjusting ocular and systemic parameters; they hypothesized that a thinner SCT is a potential risk factor for developing NAION. Although the contralateral eye was not diagnosed with NAION, some fundus changes were found. Therefore, we speculate that the abnormal blood supply in the superficial and deep layers had occurred prior to the occurrence of acute posterior ciliary arterial insufficiency and the diagnosis of NAION.

The vessel density both in superficial and deep layers was significantly lower in the uninvolved eyes compared with the healthy groups, a finding worth mentioning. However, the thickness of the superficial and deep retinal layers was not significantly different between the two groups. Compared with the normal subjects, the vessel density was significantly reduced in contralateral uninvolved eyes; however, the corresponding retinal thickness did not change. A reasonable explanation is that the vascular density decreases, reflecting that the blood supply deficiency might occur before the retinal morphologic changes. This vascular density change may be an indicator of early ocular microcirculation disorder before NAION is clinically diagnosed. The development of NAION might occur when the vascular density changes in the uninvolved eyes in NAION patients.

In the present study, the diagnostic accuracy of vessel density measurements was evaluated for differentiating NAION eyes from healthy eyes, as well as uninvolved eyes from healthy subjects. In the NAION and healthy eyes, OCT-A SVD and DVD measures have a similar diagnostic accuracy as RNFL thickness, with AUROCs ± standard error (SE) of 0.96 ± 0.03, 0.89 ± 0.04, and 0.99 ± 0.01, respectively. The whole SVD was found as a better indicator compared with the RNFL thickness for differentiating uninvolved eyes from healthy subjects, with AUROCs ± SE of 0.90 ± 0.05 and 0.68 ± 0.07, respectively. The present study was novel in using OCT-A to differentiate between uninvolved and healthy eyes.

The sample size of this study was relatively small, which may have limited the statistical strength of the analysis. Future studies should be performed with larger cohorts and longer follow-up periods to study changes of retinal choroidal structure in NAION patients. The other limitation was that OCT-A can clearly visualize each layer of retinal capillaries and microvascular status for NAION. However, OCT-A did not show clearly the structural of choroidal vessels, there were measurement errors in choroidal vascular density. Later studies require OCT-A devices with better choroidal imaging quality.

## Conclusions

OCT-A vessel density and retinal thicknesses are lower in NAION patients compared with healthy and uninvolved eyes, and the vessel density has a similar diagnostic accuracy to RNFL thickness for differentiating healthy subjects from NAION patients. It demonstrated that not only peripapillary vessel density of NAION eye had been impaired, but also macular vessel density. Vessel density might occur before retinal thickness changes at an early stage of NAION, making it a sensitive indicator in the early diagnosis of this disease.

## Additional file

Additional file 1: Raw data in this study. (XLSX 69 kb)
